# Transcription of the *var* genes from a freshly-obtained field isolate of *Plasmodium falciparum* shows more variable switching patterns than long laboratory-adapted isolates

**DOI:** 10.1186/s12936-015-0565-y

**Published:** 2015-02-07

**Authors:** Run Ye, Dongmei Zhang, Biaobang Chen, Yongqiang Zhu, Yilong Zhang, Shengyue Wang, Weiqing Pan

**Affiliations:** Department of Tropical Infectious Disease, Second Military Medical University, Shanghai, 200433 China; Shanghai-Ministry of Science and Technology Key Laboratory of Health and Disease Genomics, Chinese National Human Genome Center at Shanghai, Shanghai, 201203 China; Institute for Infectious Diseases and Vaccine Development, Tongji University School of Medicine, Shanghai, 200092 China

**Keywords:** *Plasmodium falciparum*, *var* gene families, Switching patterns, Gene expression, Transcription profiles, Wild isolate

## Abstract

**Background:**

Antigenic variation in *Plasmodium falciparum* involves switching among multicopy *var* gene family and is responsible for immune evasion and the maintenance of chronic infections. Current understanding of *var* gene expression and switching patterns comes from experiments conducted on long laboratory-adapted strains, with little known about their wild counterparts.

**Methods:**

Genome sequencing was used to obtain 50 *var* genes from a parasite isolated from the China-Myanmar border. Four clones with different dominant *var* genes were cultured *in vitro* in replicates for 50 generations. Transcription of the individual *var* gene was detected by real-time PCR and then the switching process was analysed.

**Results:**

The expression of multicopy *var* genes is mutually exclusive in clones of a wild *P. falciparum* isolate. The activation of distinct primary dominant *var* genes leads to different and favoured switching patterns in the four clones. The on/off rates of individual *var* genes are variable and the choice of subsequent dominant *var* genes are random, which results in the different switching patterns among replicates of each clonal wild *P. falciparum* isolate with near identical initial transcription profiles.

**Conclusions:**

This study suggests that the switching patterns of *var* genes are abundant, which consist of both conserved and random parts.

**Electronic supplementary material:**

The online version of this article (doi:10.1186/s12936-015-0565-y) contains supplementary material, which is available to authorized users.

## Background

*Plasmodium falciparum* is the most virulent form of the malaria species infecting humans, and is responsible for greatest mortality associated with the disease. *Plasmodium falciparum* erythrocyte membrane protein 1 (PfEMP1), expressed on the surface of infected red blood cells [1,2], plays a key role in the pathogenicity and immune evasion of *P. falciparum* [3]. PfEMP1s, encoded by the *var* multigene families, comprising an N terminal segment (NTS), variable numbers of Duffy binding-like domains (DBLα-ε), one or two cysteine-rich, inter-domain regions (CIDRα-γ), a transmembrane (TM) domain, a C2 domain, and a conserved intracellular acidic terminal segment (ATS) [4,5]. There are about 60 *var* genes in the *P. falciparum* clone 3D7 *,* although still under revision now [6], the most supported view is that each individual parasite expresses only a single *var* gene; the others are silenced in a process known as mutually exclusive expression [7,8]. Multiple means of genetic regulation mediate this process to protect the limited *var* gene repertoire [9-14].

Switching among the expression of different *var* genes allows parasites to avoid the effects of the acquired immune response generated by the host against PfEMP1 and, thus, to sustain a long-term infection. To date, *in vitro* research on *var* gene switching has been conducted either in clones or in phenotype-selected parasite lines where a dominant *var* gene is expressed. The effects of cellular memory ensure that most daughter parasites will express the same *var* gene [15-17]. This coordinates gene expression and ensures that the parasite’s repertoire of antigenic types is not rapidly exhausted.

There are three transcription states for a *var* gene: active, inactive but capable of being activated, and highly silenced [18]. The probability that a gene will be turned on or turned off is not associated with chromosomal position nor the type of promoter *per se* but rather on the intrinsic properties of each gene [16]. The initial dominant transcript determines the switch direction, while the ability to switch to particular variant types may depend on the antigenic switching history of the parasite [18-20]. Furthermore, switch rates have been suggested to be intrinsic and constant for the same *var* gene but different for individual *var* genes [18]. Switch rates have been estimated to be approximately 2% or less per generation in long-term *in vitro* cultures, but can reach 16% in hosts infected with the laboratory clone 3D7 [21,22]. Recent studies also showed that the switch on/off rate is associated with chromosomal position, with centrally located genes apparently more highly transcribed *in vitro* than those in sub-telomeric location [16,19,20,23,24], especially those that are short and highly diverse [25]. As a result of these intrinsic factors, the switching patterns of *var* genes are thought to be non-random. Indeed Recker *et al*. [26] revealed a highly structured switching pattern, consisting of an initially dominant transcript that switches via a set of switch-intermediates either to a new dominant transcript or back to the original. Similarly, Enderes *et al*. [20] also suggested the existence of an intrinsic *var* gene transcription programme that operates independently of genetic background.

The current understanding of *var* gene transcriptional regulation and switching comes from studies conducted on generations of *in vitro* long cultured laboratory-adapted parasite lines. Consequently, little is known of the processes operating especially within wild parasites. One of the difficulties with investigating wild isolates is the high sequence diversity observed among *var* genes. In addition, there is little genomic overlap of *var* genes among different *P. falciparum* isolates [27], making it difficult to determine the sequence of individual *var* genes. Despite the high diversity of *var* sequences, there are still some common structures among isolates, such as the semi-conserved head structure, consisting of NTS-DBLα-CIDR1 domains, and the single DBLα domain found in nearly each *var* gene [27-29]. Universal primers and *var* group-specific primers have been generated to amplify many DBLα sequences of field isolates and detect the transcription of *var* genes in clinical samples [24,30-34]. However, this approach is subject to error from primer bias and the over-estimation of the frequency of minor transcripts [35]. In addition, it is impossible to compare the transcription of each *var* gene quantitatively. To resolve this problem, *var* gene specific primer sets are needed (such as those recently designed for 3D7 and HB3) [25,36]. It may be possible to place the specific primers in the hypervariable regions of the DBLα domains [37], while it is first necessary to obtain sequences of the *var* gene repertoire of the wild isolates [27].

In this work, *var* gene sequences of a wild *P. falciparum* isolate were obtained by Illumina Solexa sequencing and then to investigate *var* gene switching in clonal wild *P. falciparum* isolates and compare these switching patterns with those reported for long cultured laboratory-adapted parasite isolates.

## Methods

### Parasite culture, genomic DNA extraction and genotyping

The wild isolate FCYN0906 was collected from the border area of south China (Yunnan Province) and Myanmar, and was approved by the Ethics Committee of Second Military Medical University. The patient was male and 25 years old with mild malaria. Written informed consent was obtained from the patient for the publication of this report and any accompanying images. The sample was thawed and cultivated at 3% haematocrit in RPMI 1640 medium (Invitrogen), supplemented with Albumax I (Invitrogen), hypoxanthine (Sigma) and gentamicin (Sigma). Parasites were incubated at 37°C in an atmosphere of 5% O_2_, 5% CO_2_, and 90% N_2_. Clones were obtained from the wild isolate by limiting dilution [38] (20 reinvasions passed since the isolate was taken from the patient). DNA was isolated using the QIAamp Mini Kit (Qiagen) and clones 4C, 4H, 5H, 6G were genotyped according to the DNA fragment length of eight microsatellite alleles (ARA2, TA1, TA60, TA81, TA87, TA109, Pfpk2, Polyα) as described [39].

### Solexa sequencing, bio-informatic analysis and assembling of the *var* genes

Genomic DNA of clone FCYN0906-5H was prepared for the solexa sequencing. Two × 100 bp paired-end sequencing was done on a single lane of the Illumina HiSeq2000 following standard protocols. In total, 10,259,515 paired end reads were generated and the average coverage was 90×. The contigs were assembled by Velvet (version 1.2.03) and blasted with the PfEMP-1 protein sequence database in NCBI. All the contigs belong to *var* genes were predicted in VarDom 1.0 Service. A total of 66 sequences were found to correlate with DBLα, of which 47 had the 5′DIGDI and 3′PQFLR consensus sequences. Nineteen primers were designed to complete the DBLα domains combined with primers DBLα-AF/BR [30] through PCR and sequencing. Finally, 50 sequences (accession numbers: KJ856447-KJ856496) with DBLα domains were affirmed that present the *var* genes of the wild isolate individually. The 50 *var* genes were sub-grouped according to the upstream sequences blasted with the 5′ regions of 60 *var* genes in clone 3D7. Primers upsB-5′UTR and upsC-5′UTR [32] previous described were also helpful to identify three upsB subtype (*var149*, *var25*, *var46*) and four upsC subtype (*var131*, *var163*, *var170*, *var51*) *var* genes, respectively. Distance trees were constructed by the p-distance/neighbour-joining (NJ) method with 1,000 times bootstrap using MEGA version 4. Observed clusters from each tree were confirmed visually on alignments.

### Sample preparation, RNA extraction and cDNA synthesis

Clone 4C, 4H, 5H, and 6G were thawed and the first transcriptional analysis was undertaken to identify the dominant *var* genes for each clone (30 reinvasions passed since the isolate was taken from the patient). The culture was then divided into two biological replicates and the transcriptional profiling was observed for 50 generations. Twelve-18-hour synchronic ring-stage parasites were harvested every ten generations. Then the four clones were thawed again and the whole process repeated. RNA was extracted using Trizol (Invitrogen) according to the manufacturer’s instructions and treated with Amplification Grade DNase I (Sigma) to remove the potential contamination of genomic DNA. cDNA was synthesized from 800 ng of RNA with random hexamers and oligo(dT) primers in a 30-μl reaction according to the manufacturer’s recommendations (Takara).

### Primer design and quantitative real-time PCR

Fifty *var* gene-specific primer sets were designed depending on the hypervariable regions of the DBLα using primer 5 (see Additional file 1). All primer pairs were first tested on genomic DNA, PCR started with heating on 94°C for 5 min, followed by 30 cycles of 94°C for 30 sec, 60°C for 30 sec and 65°C for 20 sec. Final elongation was performed for 10 min at 65°C; the PCR products were sequenced and compared with the *var* gene sequences in MEGA4 to make sure the specificity of the primers. Then the amplification efficiencies of the primers were tested on ten-fold dilutions of genomic DNA. Specificity of amplification was ascertained by melting-curve analysis of each PCR product. Quantitative amplification was performed at a final primer concentration of 0.4 μM using Takara SYBR Premix Ex TaqTM in 15-μl reactions. All runs were done in triplicate under the condition 95°C 30 sec, followed by 40 cycles (95°C for 5 sec and 60°C for 30 sec) on the Roche LightCycler ®480II machine. Primer pairs varied >1.5 threshold cycle (Ct) values from the median or had effciency value (Amplifcation) < 1.85 were redesigned. The condition of the reaction was same for the cDNA. Specificity of amplification was ascertained by melting-curve analysis of each PCR product. Seryl-tRNA synthetase and fructose biphosphate aldolase were used as endogenous control genes. The ΔCt for each individual primer pair was calculated by subtracting the measured Ct value from the Ct value of the control seryl-tRNA synthetase. Relative copy numbers were obtained with the formula 2^-ΔCt^.

### Calculation of on and off rates for individual *var* loci

The switching on/off rates were calculated as previously described [19]: the increase or decrease of the expression of the *var* gene between two time points divided by the number of generations between these two time points.

## Results

### Identification of 50 *var* genes in the genome of the wild *Plasmodium falciparum* isolate

**Figure 1:**
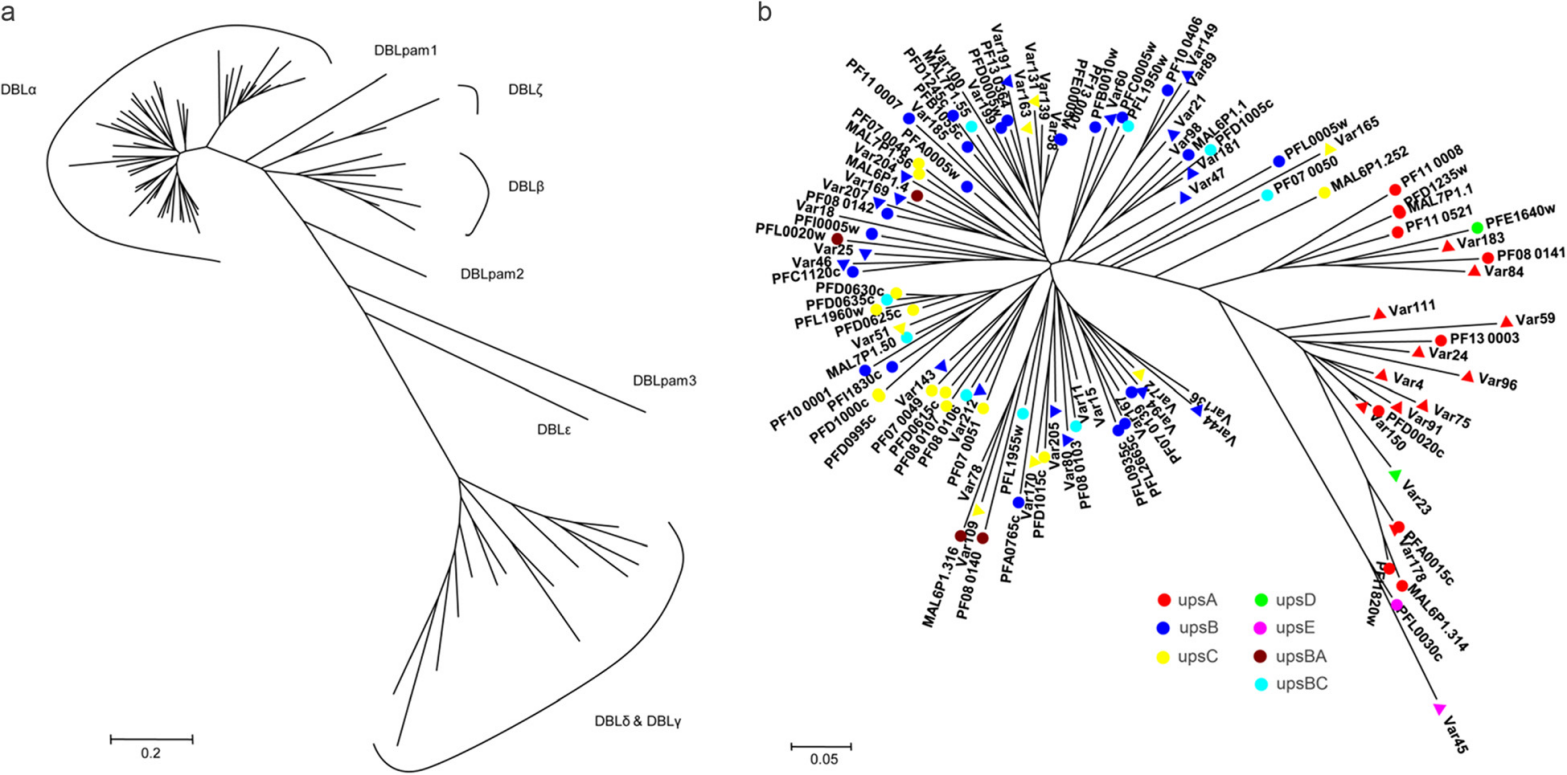
**Subclassification of DBL domains of FCYN0906-5H. a**. Distance tree of DBL sequences of FCYN0906-5H generated using the p-distance/NJ method. DBL domains are divided into six major groups and the DBLα branch separated from the other groups. **b**. Phylogenetic trees of all DBLα amino acid sequences between the laboratory clone (3D7) and the wild isolate(FCYN0906-5H). The *var* genes of 3D7 are labelled as circles and those of FCYN0906-5H are labelled as triangles. *Var* gene groups are marked by different colours. Although upsB and upsC sub-groups overlapped, the upsA sub-group could be distinguished from others. Meanwhile, *var4* and *var84* had no upstream sequences but were assumed to be a sub-type of upsA as they were also within the upsA sub-group.

**Figure 2:**
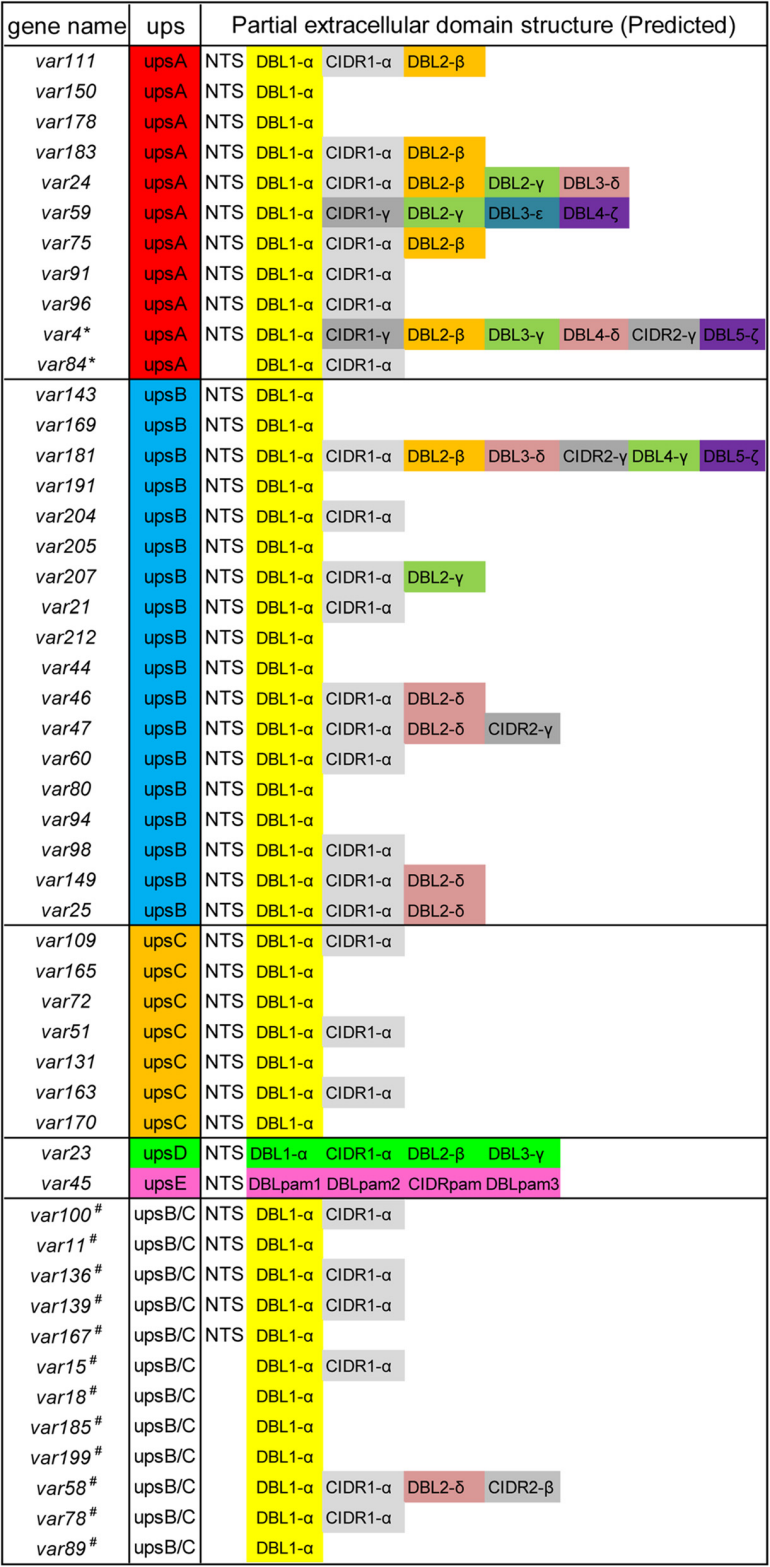
**Schematic diagram of the repertoire of** ***var*** **genes of the wild** ***Plasmodium*** **isolate, FCYN0906-5H.** The gene names, Ups sequence type and domain architecture are listed. Where known, the *var* genes have been grouped according to 5′ flanking sequence (Ups type). The extracellular domains of PfEMP1 have been classified by sequence criteria. * *var4* and *var84* had no upstream sequences but had DBLα sequences of group A. # indicates *var* genes that had no upstream sequences but belong to either group B or group C according to the DBLα amino acid sequences.

Four clones from the wild *P. falciparum* isolate FCYN0906 were obtained by limiting dilution (designated as FCYN0906-4C, 4H, 5H, and 6G), which exhibited identical genotypes when analysed with eight microsatellites (see Additional file 2). One clone (FCYN0906-5H) genome was sequenced and the reads were assembled into contigs to obtain 50 partial *var* gene sequences (see Methods for more details). These 50 partial *var* gene sequences represented the 50 *var* genes of the wild isolate because each sequence had a DBLα domain that was separated from DBL(β-ζ) (Figure 1a). In addition, 42 sequences possessed an NTS domain. A total of 38 *var* genes were sub-grouped according to their 5′ flanking sequence (50–500 bp) and the phylogenetic analysis of DBLα sequences (Figure 1b). Eleven upsA, 18 upsB, seven upsC, one upsD, one upsE *var* genes were identified, while the remaining 12 genes belonged to either the upsB or upsC sub-group (Figure 2).

### Mutually exclusive expression of *var* genes in clonal wild isolates

**Figure 3:**
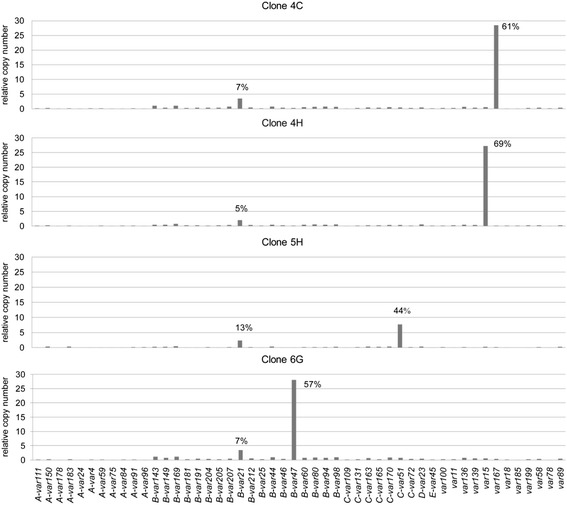
**Histograms showing the mutually exclusive expression of** ***var*** **genes in the four clones.** The four clones expressed different dominant *var* genes which were present at proportions ranging from 44 to 69%. There were also minor transcripts with *var21* as the second dominant *var* gene across all four clones.

Fifty *var* gene-specific primer sets were designed depending on the hypervariable regions of the DBLα sequences (see Additional file 1). The *var* gene expression of four clones (FCYN0906-4C, 4H, 5H, 6G) was measured as soon as the culture reached the required parasitaemia (2-5%). Real-time PCR results showed that all four clones had different dominant *var* genes, of which the proportion of the total signal was 61, 69, 44 and 57%, respectively (Figure 3). Interestingly, *var21* (upsB group) was the second dominant *var* gene among all four clones. So despite the presence of minor transcripts, the allelic exclusion of *var* genes was evident in this wild isolate, just as in long laboratory-adapted clone 3D7.

### The *var* gene switching patterns of the four clones

**Figure 4:**
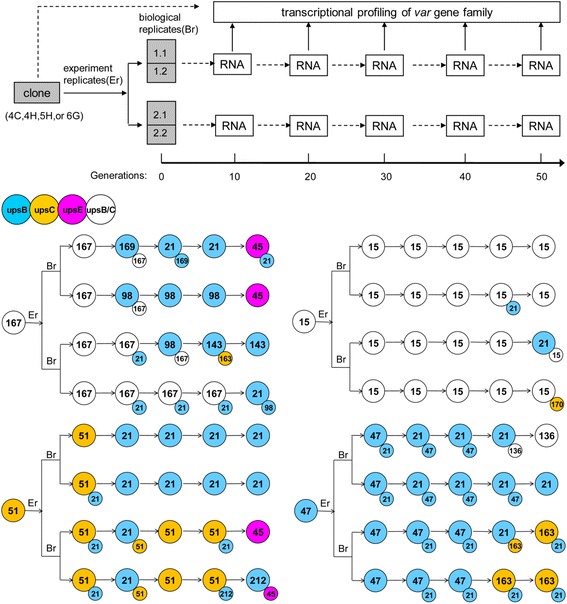
**Diagram depicting the variable** ***var*** **gene switching patterns of the four clones and the replicates.** The second dominant *var* gene was identified when its transcription level reached 40% of that of the dominant *var* gene. Clone 4C: *var167* switches to *var21* in 4C-A, 4C-D, *var143* in 4C-C, and *var98* in 4C-B. However, at the last time point, the dominant *var* gene in 4C-A, 4C-B, both switch to *var45*. Clone 4H: *var15* is maintained for over 50 generations in 4H-A, 4H-B, however, its expression falls in 4H-C, 4H-D, switches to *var21* in 4H-C. Clone5H: *var51* switches off quickly and immediately switches to *var21* in 5H-A, 5H-B, but in 5H-C, 5H-D, *var51* slows down the off rate and switches to *var45*, *var212*. Clone 6G: *var47* switches to *var136* in 6G-A and *var21* in 6G-B. The change in the transcriptional trend is similar in 6G-C and 6G-D; *var47* switches to *var163* gradually. *var51* and *var47* were predicted to be upsC and upsB group individually, *var167* and *var15* should belong to either upsB or upsC but not upsA group from the phylogenetic trees of all *var*-DBLα-contig amino acid sequences of the wild isolate.

According to the results of Enderes *et al*. [20], *var* gene switching patterns occur via a conserved switching programme. Thus each clone culture was divided into two replicates to determine whether these near-identical replicates exhibited the same switching pattern. For the next 50 generations, ring-stage parasites were harvested every ten generations and the transcription level of the *var* genes measured by real-time PCR with 50 gene-specific primer sets; then the whole process was repeated. Results showed different switching patterns among the four clones (see Additional file 3). Surprisingly, switching directions were also various within the four replicates of each clone, which was contrary to those reported in previous studies [20]. The dominant transcripts were often upsB or upsC *var* genes and the upsA *var* genes of the wild isolate were rarely activated during the experiment. Figure 4 shows the stages in the switching of the dominant transcript. The reproducibility of data is further evidenced in Additional file 4.

### Expression of distinct dominant *var* genes among the four clones

Previous work has suggested that the activation of different *var* loci in an isogenic background results in different switching patterns [18-20]. Hence, the ability to switch to particular variants might depend on the antigenic switching history of the parasite. In this study, except the more active *var21* and *var45*, each clone favoured particular *var* genes that were not expressed strongly in the other clones (e.g., *var147*, *var98*, *var143* in clone 4C, *var15* in clone 4H, *var51*, *var212* in clone 5H, *var47*, *var163*, *var136* in clone 6G). For this reason, the initial *var* gene transcription level of the four clones were compared. At the first time point, the initial transcription level was similar across the four replicates of every clone (see Additional file 3). This suggests that the four clones differed primarily in their expression of distinct dominant *var* genes, which led to different and favoured switching patterns, consistent with previous findings. However, this suggestion cannot explain the variety of switching patterns observed among the replicates of the same clone, with near-exact initial transcription profiles.

### Switching on/off rates of a *var* gene are variable

Previous work has shown that on/off rates vary dramatically among different *var* genes, while in isogenic clones that express the same *var* gene on/off rates are constant and appear to be an intrinsic property of that particular gene. However, results of this study showed that for a given *var* gene, the switching on/off rates were not always the same and could be activated and silenced at high speed or just with a slow process (Table 1). Furthermore, *var51* even switched back in 5H-C and 5H-D although it switched off quickly in 5H-A and 5H-B. A greater number of switching patterns were observed since the dominant *var* gene could persist for a long time, switch back, switch to an alternative dominant *var* gene slowly or immediately. Indeed, this was an important factor mediating the variety of switching directions among the replicates. In accordance with previous findings, there were also some *var* genes that exhibited constant switching on/off rates in different clones or replicates over 20 generations (see Additional file 5).

**Table 1:** **Variability in on/off rates of the** ***var*** **genes over 20 generations**

		**Fast**			**Slow**		
**On/off rate**	***Var*** **gene**	**Clone**	**Change of proportional** ***var*** transcript levels*	**Mean rate**	**Clone**	**Change of proportional** ***var*** transcript levels*	**Mean rate**
On rate	*var98*	4C-B	2%-29%-42%	2%	4C-D	2.3%-8%-11%	0.44%
	*var45*	5H-C	1%-6%-57%	2.8%	5H-D	0.14%-0.26%-2.24%	0.11%
	*var136*	6G-A	2%-16%-48%	2.3%	6G-D	1%-1%-1.7%	0.04%
	*var21*	5H-B	13%-14%-74%	3.05%	5H-C	11%-17%-25%	0.7%
Off rate	*var98*	4C-B	42%-32%-11%	1.55%	5H-D	2.5%-1.5%-1.3%	0.06%
	*var51*	5H-A	44%-33%-3%	2.05%	5H-B	5.2%-0.8%-0.1%	0.26%
	*var167*	4C-C	44%-18%-14%	1.5%	6G-D	1.5%-0.9%-0.5%	0.05%
	*var47*	6G-B	34%-20%-19%	0.75%	4C-C	0.9%-0.5%-0.4%	0.03%

*Var* genes can switch on/off quickly or slowly. The mean on/off rates were calculated according to the change of proportional *var* transcript levels over the 20 generations.

*during 20 generations.

### Switching patterns

When the dominant *var* gene switch off slowly, the switching patterns of the two replicates that derived from the same culture were compared. Changes of the transcription profiles were similar, especially within the first three time points (30 generations after division). For example, when the *var167* switched off, *var98*, *var21*, *var143* switched on preferentially in both replicates 4C-C and 4C-D, suggesting a conserved switching process. However, the choice of subsequent dominant *var* gene appeared to be random so that the switch directions of the two replicates changed. This phenomenon was confirmed in the other clones (such as 5H-C and 5H-D, 6G-A and 6G-B). Hence, switch patterns could consist of both conserved and random processes.

## Discussion

Studies on gene expression in the *P. falciparum* clone 3D7 have shown that the mutually exclusive expression of virulence genes is used by the parasite to slow the depletion of the limited number of genes contained in the multicopy *var* gene family [7,8]. However, it is still unknown whether the same strategy is used in other isolates. In this study, a wild isolate was chosen and the transcription levels of the entire *var* gene repertoire are quantitative and comparable. The results showed that each of the initial clones exhibited a distinct dominant *var* gene and then could even reach 90% of the total signal, which confirm the allelic exclusion of *var* genes in this wild isolate.

Previous studies about the clinical *P. falciparum* isolates have shown that during the *in vitro* adaption of the parasites, the transcription of *var* genes changed a lot. The ups A *var* genes declined and upsB and upsC *var* genes were activated frequently [24,34,37]. The *var* genes in this wild isolate could be found as both active and inactive types and most upsA *var* genes were silenced and rarely activated. In particular, *var21* was more active in all the four clones and it was clearly recognized by the mutually exclusive expression system as in clone 5H. Thus like *var27* and *var29* that were preferentially expressed in the laboratory strain HB3 [25], *var21* is the gene preferred by isolate FCYN0906.

The switching of *var* genes has been extensively studied in clone 3D7. Horrocks *et al*. [18] first noted that some transitions appear to be disallowed depending on the recent variant antigen expression history of the parasite clone. Similarly, Enderes *et al*. [20] also found that the last active *var* locus has an influence on subsequent *var* gene activation. In this study, the four clones had different initial dominant *var* genes. The following switching patterns and the subsequent activated *var* genes seemed to be favoured by each clone, which was in agreement with previous studies.

Previous research has suggested that the rate at which the individual *var* genes become transcriptionally activated or silenced (on/off rates) are particular to individual genes and relatively stable over time [18,19]. However, the results of this study indicated that the on/off rates of *var* genes in the wild isolate were highly variable. As there were also some *var* genes with constant on/off rates, it is possible that variable rates also exist among the laboratory strains, but Horrocks happened to investigate *var* genes that exhibited constant on/off rates. Recker *et al*. [26] showed that simple differences in switch rates could not explain their switching data, and instead proposed a mechanism of biased switching to explain the very high on rates and very low off rates they observed. Their best model, however, still assumed that the switch rates and switch biases themselves were constant. Although the model is improved continuously [25,40], if the switch rates and switch biases of individual *var* genes were intrinsic and constant, then the replicates with near-identical initial transcription profiles would be expected to have the same switching pattern, but not for all four clones. In other studies, however, *var* genes have been found to switch rapidly once the first gene has been expressed, with subsequent switching occurring at a much lower rate [22], and switching rates can be much higher in individual clinical isolates [34] which may be influenced by the physiological states of the patients. The variability of the switch rates of *var* genes could also be an explanation of the high on rates and very low off rates generally observed. The advantage of this would be that it ensures that the dominant *var* gene is maintained when it is optimal for the infection, but allows it to change rapidly once it is recognized by the immune system. A recent study showed that disrupting PfSET2/RNA pol II interactions in transgenic parasites induced rapid *var* gene expression switching [41]. The ability of the wild isolate to accelerate the *var* gene expression switching needs to be further studied.

Frank *et al*. [19] indicated that despite the long-term bias towards expression of *var* genes with low off rates, there was no predetermined order of *var* gene expression that ensures the generation of heterogeneous *var* gene expression patterns. However, in their study, transcription levels were monitored at long intervals, while close attention was only paid to the particular *var* genes to which each clone switched. Enderes *et al*. [20] suggests the existence of an intrinsic *var* gene transcription process that occurs independently of genetic background. However, in that study, due to epigenetic memory, there were no switches of the dominant *var* genes in transgenic parasites and filed parasites during the whole experiment. The same conclusion would be reached if observations had stopped at 30 generations after the division. In fact, continuing culture of the isolates revealed that the following dominant transcripts were diverse and chosen randomly, leading to different patterns. Moreover, when the following dominant *var* genes happened to be identical, the two replicates like 6G-C and 6G-D still exhibited the same switching direction.

## Conclusions

The data of this study show that the expression of multicopy *var* genes is mutually exclusive in clones of a wild *P. falciparum* isolate, that the *var* genes on/off rates are variable, and that the dominant *var* gene can persist for a long time, or switch back, or switch either slowly or immediately to an alternative dominant *var* gene. When the initial dominant *var* gene is maintained, or is gradually reduced, variation in transcription levels follows a conserved switching process. When the expression of the dominant *var* genes falls it is accompanied by the switching on of other dominant *var* genes that appear to be expressed at random. The data reveal that switching patterns are not solely the result of an intrinsic, conserved process, but a result of both conserved and random processes. Hence, the complete switching process of multicopy *var* genes is more complex than previously described.

## Additional files

Additional file 1:
**The 50** ***var*** **gene specific primer sets used in the real-time PCR assays.**
Additional file 2:
**Allele length (bp) of the four clones at eight microsatellite loci.**
Additional file 3:
**(a-d) Transcription levels of the entire** ***var*** **gene family in the four clones across biological replicates.** The transcription profiles of the *var* genes were measured every ten generations during the culture of 50 generations. (G: generations). Additional file 4:
**Replicate transcript levels of 4C-D and 6G-C measured at 30 generations after division.** The standard deviations are shown as error bars. Additional file 5:
**The** ***var*** **genes that switched on/off at a constant rate.** The transcriptional level of the four *var* genes were low during the culture of 20 generations and the switch on/off rates were constant for the same *var* gene in different clones.
