# Supplementary material for: Transcription of the var genes from a freshly-obtained field isolate of Plasmodium falciparum shows more variable switching patterns than long laboratory-adapted isolates
Source: Malar J. 2015 Feb 7;14:66. doi: 10.1186/s12936-015-0565-y (PMC4332720; doi:10.1186/s12936-015-0565-y)
Supplement: Additional file 2: — Allele length (bp) of the four clones at eight microsatellite loci. [file 12936_2015_565_MOESM2_ESM.docx]

**Additional file 2**. **Allele length (bp) of the four clones at 8 microsatellite loci.**

| Clone | ARA2 | TA1 | TA60 | TA81 | TA87 | TA109 | Pfpk2 | Polyα |
| --- | --- | --- | --- | --- | --- | --- | --- | --- |
| 4C | 65 | 167.3 | 78.9 | 122.6 | 111 | 163.2 | 158.1 | 161.8 |
| 4H | 65.5 | 167.4 | 78.9 | 122.4 | 111 | 163.5 | 157.6 | 161.1 |
| 5H | 65.2 | 167.5 | 79 | 122.3 | 111.2 | 163.6 | 157.5 | 161.8 |
| 6G | 65.2 | 167.2 | 79.1 | 122.4 | 111.1 | 163.3 | 157.4 | 161.5 |
