# Supplementary figures and images for: Transcription of the var genes from a freshly-obtained field isolate of Plasmodium falciparum shows more variable switching patterns than long laboratory-adapted isolates
Source: Malar J. 2015 Feb 7;14:66. doi: 10.1186/s12936-015-0565-y (PMC4332720; doi:10.1186/s12936-015-0565-y)

a

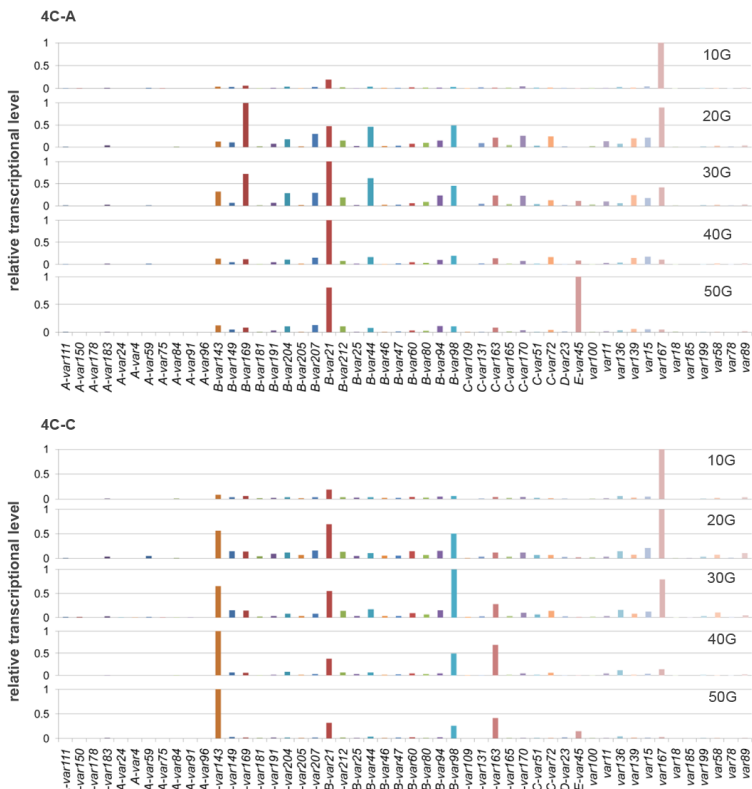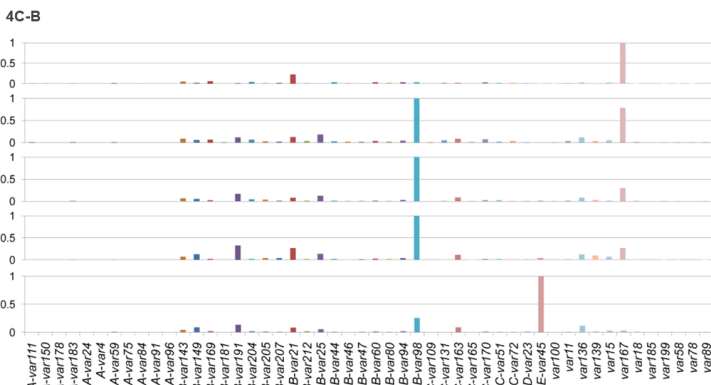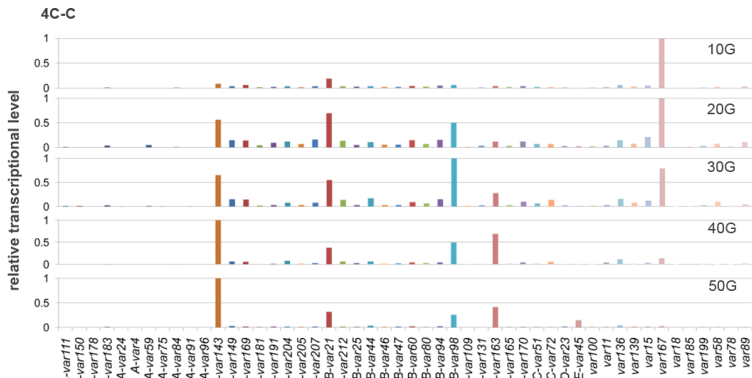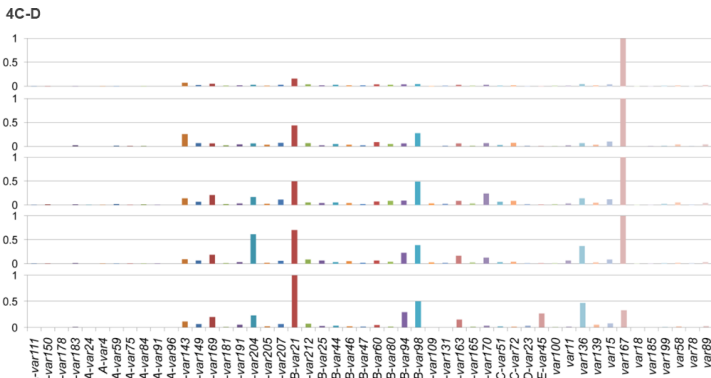

b

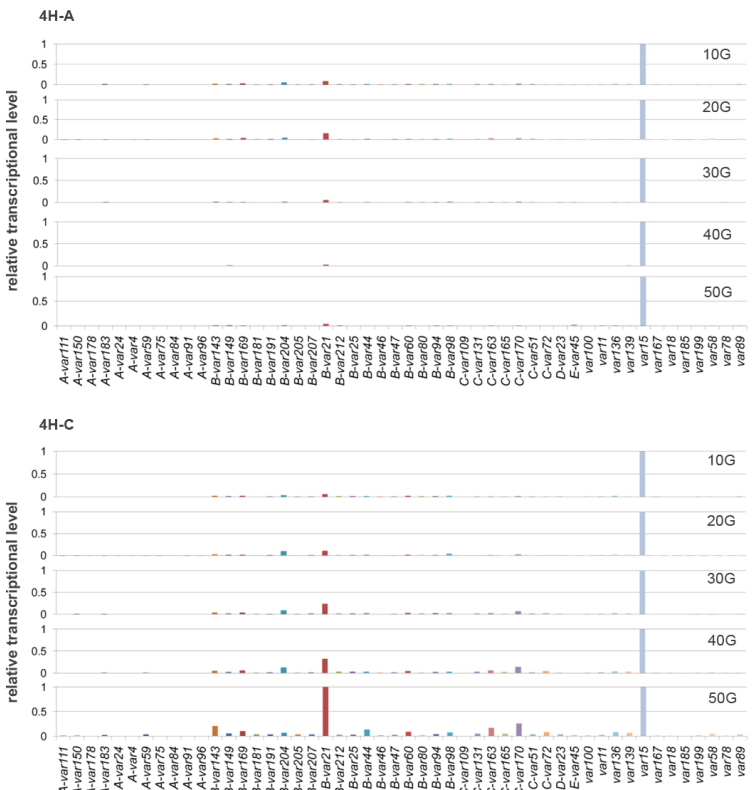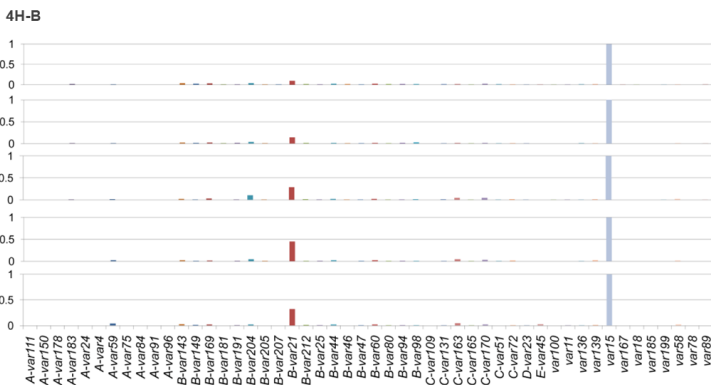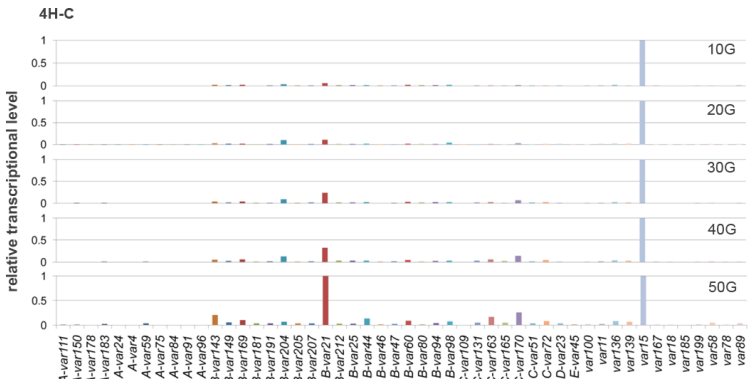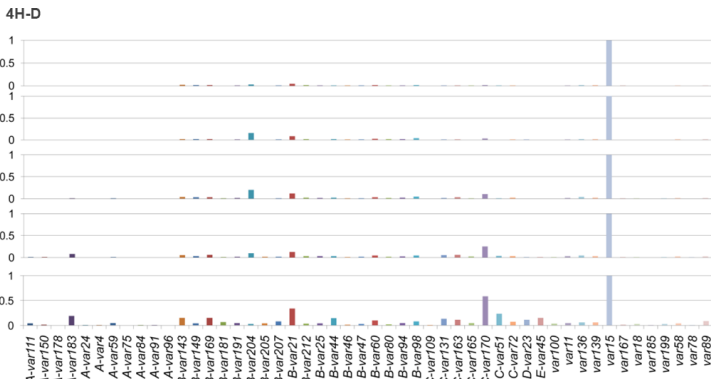

C

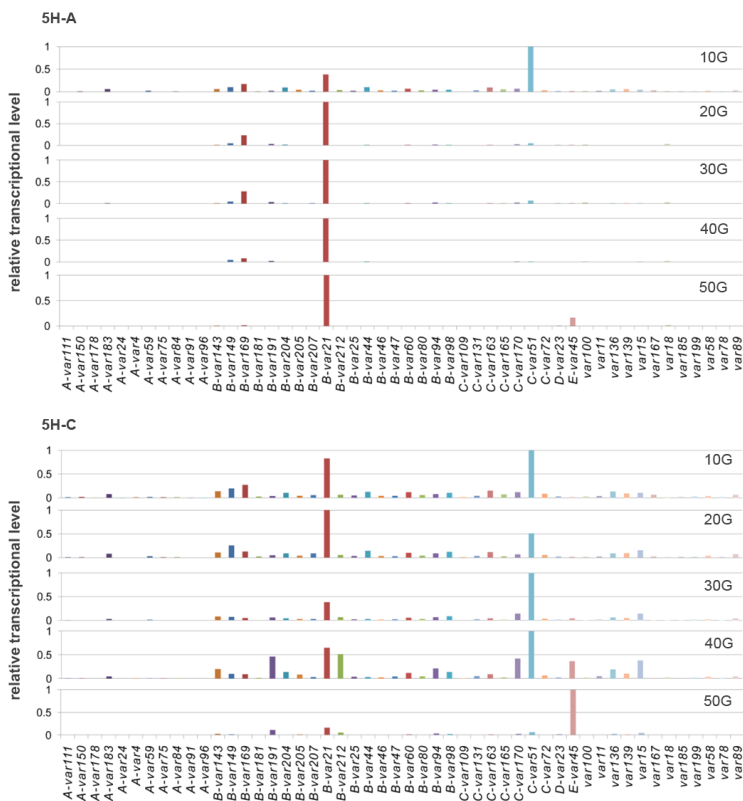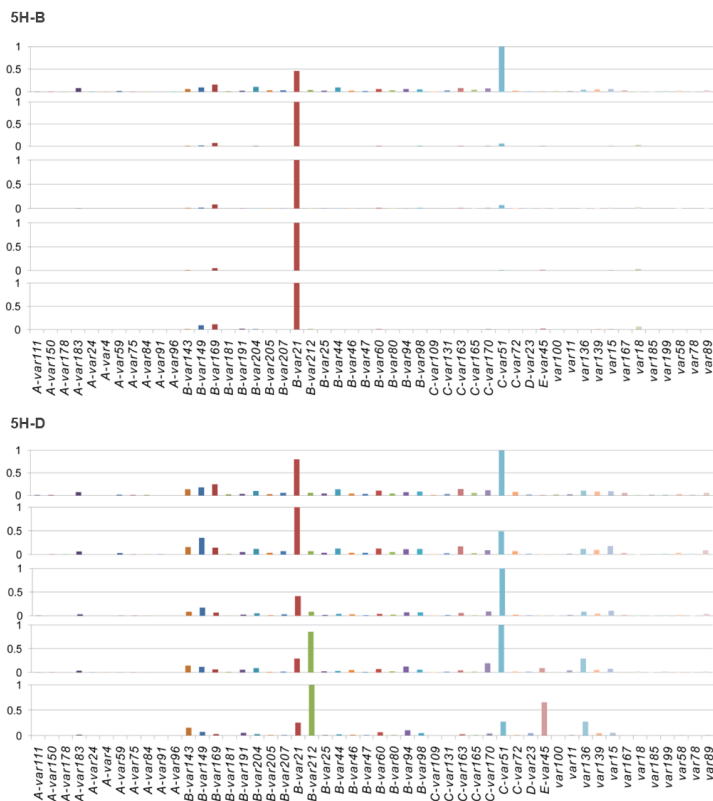

d

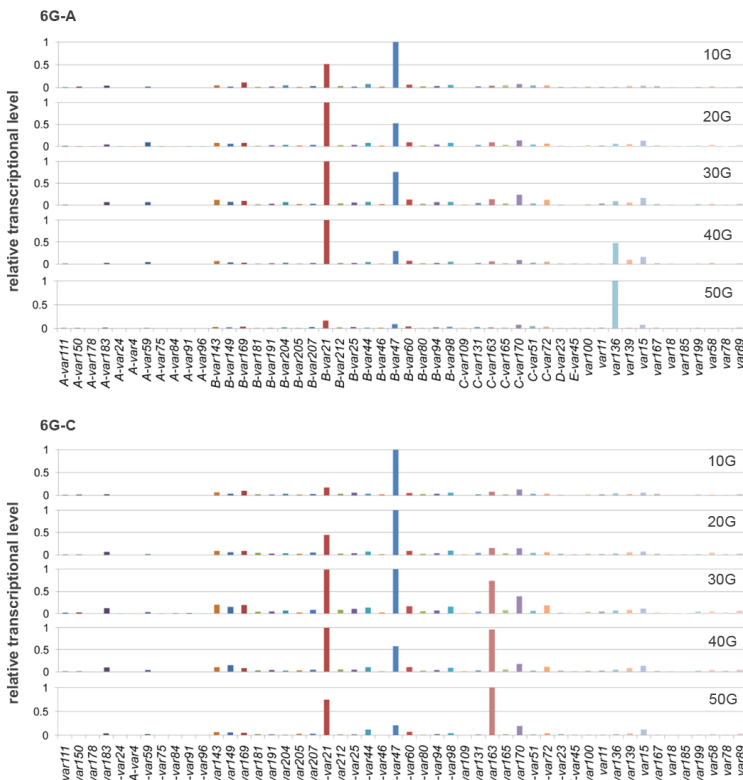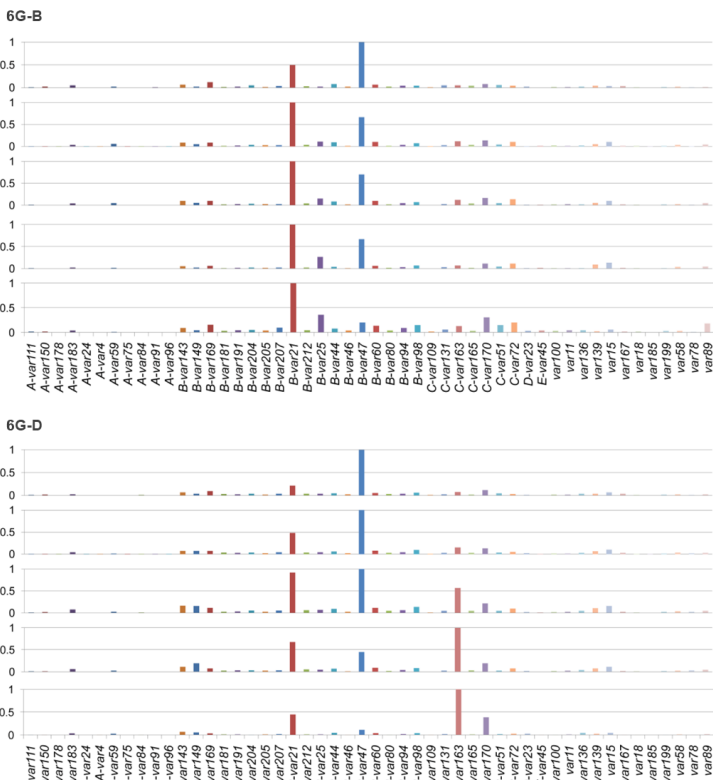

Supplement: Additional file 3: — (a-d) Transcription levels of the entire var gene family in the four clones across biological replicates. The transcription profiles of the var genes were measured every ten generations during the culture of 50 generations. (G: generations). [file 12936_2015_565_MOESM3_ESM.pdf]

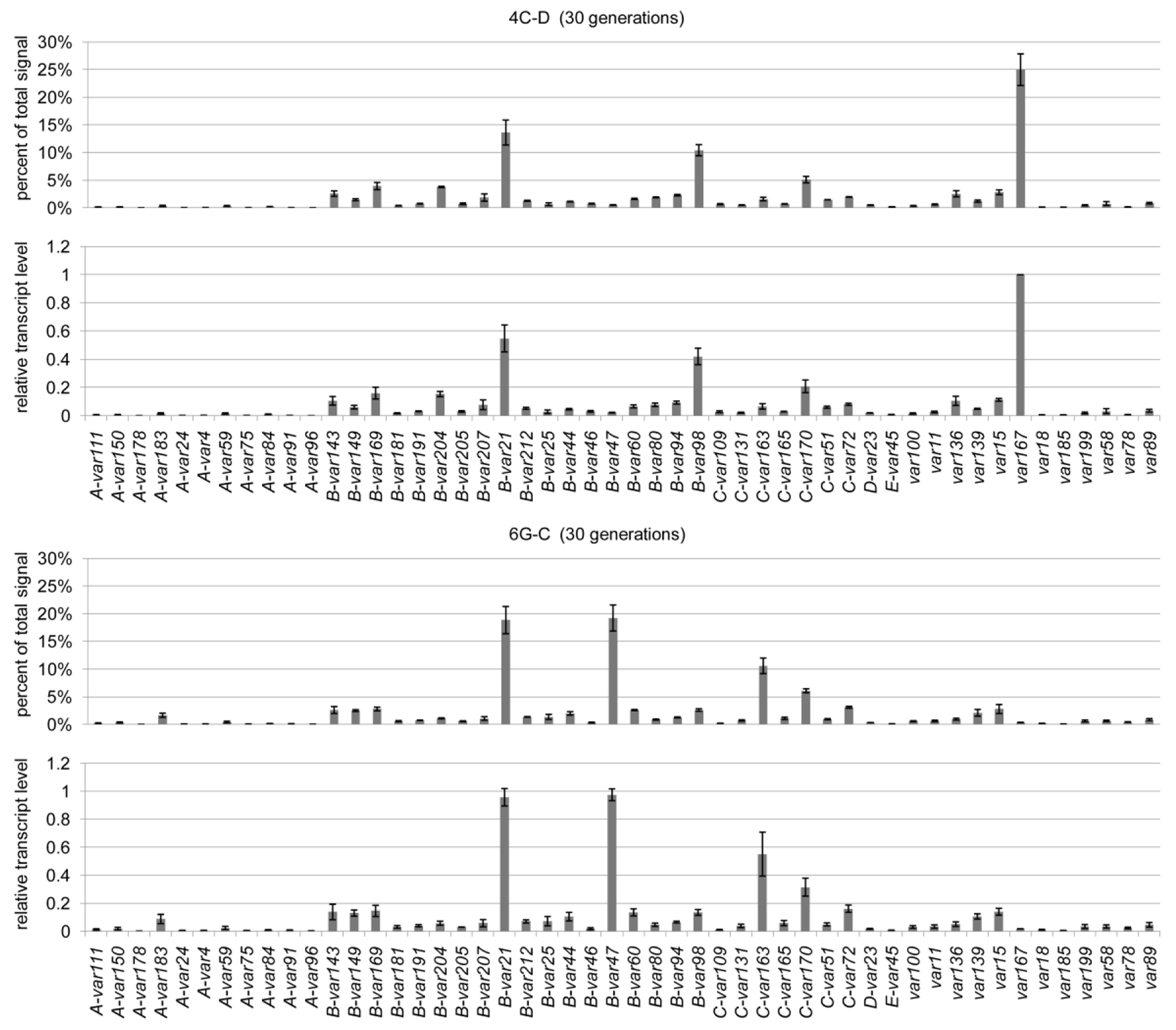

Supplement: Additional file 4: — Replicate transcript levels of 4C-D and 6G-C measured at 30 generations after division. The standard deviations are shown as error bars. [file 12936_2015_565_MOESM4_ESM.tiff]
