# Supplementary material for: Transcription of the var genes from a freshly-obtained field isolate of Plasmodium falciparum shows more variable switching patterns than long laboratory-adapted isolates
Source: Malar J. 2015 Feb 7;14:66. doi: 10.1186/s12936-015-0565-y (PMC4332720; doi:10.1186/s12936-015-0565-y)
Supplement: Additional file 5: — The var genes that switched on/off at a constant rate. The transcriptional level of the four var genes were low during the culture of 20 generations and the switch on/off rates were constant for the same var gene in different clones. [file 12936_2015_565_MOESM5_ESM.docx]

| on/off rate | *var* gene | | clone | Change of proportional  var transcript levels* | mean rate |  |
| --- | --- | --- | --- | --- | --- | --- |
| on rate | | *var149* | 4C-B | 1.8%-2.02%-4.1% | 0.12% |  |
|  | |  | 6G-C | 1.76%-2.54%-3.24% | 0.07% |  |
|  | | *var139* | 4C-A | 1.1%-1.2%-3.32% | 0.11% |  |
|  | |  | 6G-A | 1.44%-1.51%-3.17% | 0.09% |  |
|  | |  |  |  |  |  |
| off rate | | *var98* | 6G-D | 2.83%-2.02%-1.53% | 0.07% |  |
|  | |  | 5H-D | 2.51%-1.49%-1.34% | 0.06% |  |
|  | | *var44* | 4C-B | 0.95%-0.7%-0.58% | 0.02% |  |
|  | |  | 5H-C | 1.05%-0.49%-0.26% | 0.04% |  |

**Additional file 5. The *var* genes that switched on/off at a constant rate.**

*during 20 generations
